# Pandemic COVID-19 Joins History’s Pandemic Legion

**DOI:** 10.1128/mBio.00812-20

**Published:** 2020-05-29

**Authors:** David M. Morens, Peter Daszak, Howard Markel, Jeffery K. Taubenberger

**Affiliations:** a Office of the Director, National Institute of Allergy and Infectious Diseases, Bethesda, Maryland, USA; b Viral Pathogenesis and Evolution Section, Laboratory of Infectious Diseases, National Institute of Allergy and Infectious Diseases, Bethesda, Maryland, USA; c EcoHealth Alliance, New York, New York, USA; d Center for the History of Medicine, The University of Michigan Medical School, Ann Arbor, Michigan, USA; Icahn School of Medicine at Mount Sinai; Albert Einstein College of Medicine

**Keywords:** COVID-19, pandemic, influenza, plague

## Abstract

With great apprehension, the world is now watching the birth of a novel pandemic already causing tremendous suffering, death, and disruption of normal life. Uncertainty and dread are exacerbated by the belief that what we are experiencing is new and mysterious. However, deadly pandemics and disease emergences are not new phenomena: they have been challenging human existence throughout recorded history. Some have killed sizeable percentages of humanity, but humans have always searched for, and often found, ways of mitigating their deadly effects. We here review the ancient and modern histories of such diseases, discuss factors associated with their emergences, and attempt to identify lessons that will help us meet the current challenge.

## Abstract (precis)

With great apprehension, the world is now watching the birth of a novel pandemic already causing tremendous suffering, death, and disruption of normal life. Uncertainty and dread are exacerbated by the belief that what we are experiencing is new and mysterious. However, deadly pandemics and disease emergences are not new phenomena: they have been challenging human existence throughout recorded history. Some have killed sizeable percentages of humanity, but humans have always searched for, and often found, ways of mitigating their deadly effects.

## PERSPECTIVE

Since December 2019, the world has watched the slow-motion birth and rapid growth of a new pandemic disease, coronavirus (CoV) disease 2019 (COVID-19). As in Albert Camus’ *La Peste* (*The Plague*) (1), the familiar rhythms of our very real lives have been shaken by an unfamiliar existential threat. Rising death and case numbers have led to changes in every aspect of our work, school, recreation, travel, economic well-being, and interactions with friends and family. Among the first pandemics in the age of social media, misinformation continues to crowd out important facts, which heightens anxiety and confusion.

However, ours is hardly the only era to face such tribulations. Deadly pandemics and large-scale epidemics have challenged human existence throughout history (Table 1) (2). While these crises were once separated by centuries, or at least many decades, they are now becoming much more common. Since 2003, we have experienced severe acute respiratory syndrome (SARS) (a near pandemic), an influenza pandemic (H1N1pdm in 2009), a chikungunya pandemic (2014), a Zika pandemic (2015), and a widespread pandemic-like extension of Ebola over five African countries, with cases exported globally (2014 to 2015). Although the meaning of the word “pandemic” has recently been reinterpreted according to differing agendas (3, 4), it seems clear that we now live in an era of pandemics, newly emerging infectious diseases, and the return of old contagious foes.

**TABLE 1:** Some notable pandemic and epidemic diseases^*a*^

Yr(s)	Disease (agent)	No. of deaths	Comments
430 BCE	Plague of Athens	∼100,000	First identified transregional pandemic
541	Plague of Justinian (Yersinia pestis)	30–50 million	Pandemic; killed half of the world’s population
1340s	Black Death (Yersinia pestis)	∼50 million	Pandemic; killed at least a quarter of the world’s population
1494	Syphilis (Treponema pallidum)	>50,000	Pandemic brought to Europe from the Americas
ca. 1500	Tuberculosis	High millions	Ancient disease; became pandemic in Middle Ages
1520	*Hueyzahuatl* (Variola major)	3.5 million	Pandemic brought to the New World by Europeans
1793–1798	American plague	∼25,000	Yellow fever, which terrorized colonial America
1832	2nd cholera pandemic in Paris	18,402	Spread from India to Europe/Western Hemisphere
1918	Spanish influenza	∼50 million	Led to additional pandemics in 1957, 1968, 2009
1976–2020	Ebola	15,258	First recognized in 1976; 29 regional epidemics to date
1981	Acute hemorrhagic conjunctivitis	Few	First recognized in 1969; pandemic in 1981
1981	HIV/AIDS	∼32 million	First recognized in 1981; ongoing pandemic
2002	SARS	813	Near pandemic
2009	H1N1 swine flu	284,000	5th influenza pandemic of the century
2014	Chikungunya	Few	Pandemic, mosquito borne
2015	Zika	∼1,000?^*b*^	Pandemic, mosquito borne

a Refer to the pandemic and epidemic definitions in the text and cited references, particularly references 7 and 8. The table is not comprehensive but lists notable emergences of historical importance. Many of these diseases have emerged/reemerged on multiple occasions. For most historical pandemics, estimated numbers of deaths have varied widely, and figures cannot be considered accurate.

b Zika deaths occur mostly *in utero* or in newborns; death in older children and adults is rare.

## EARLY PANDEMIC HISTORY

**FIG 1:**
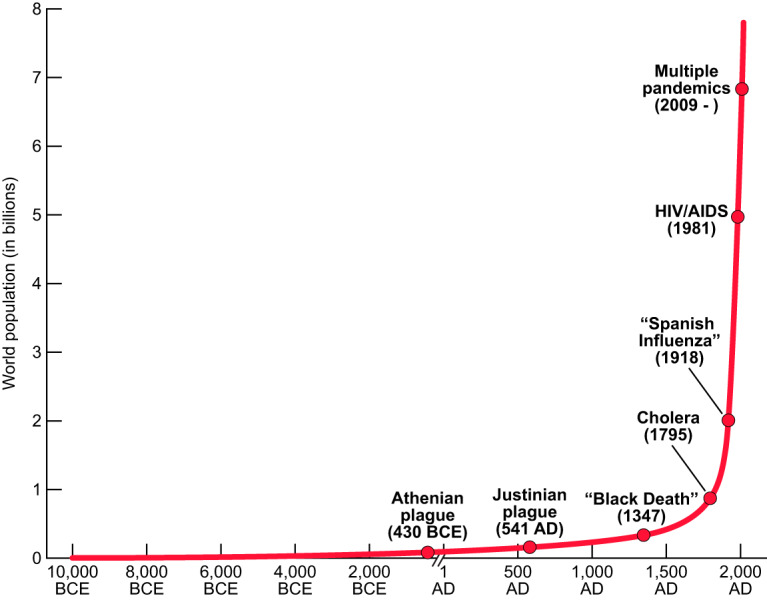
Estimated world population and selected known pandemics/widespread disease emergences, from 10,000 BCE to 2020 AD.

**FIG 2:**
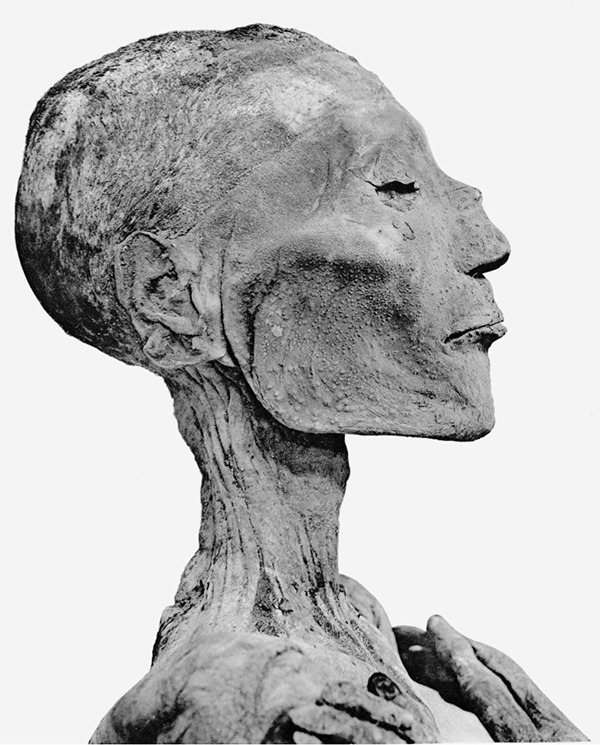
Mummy of Pharaoh Usermaatre Sekheperenre Ramesses V (ca. 1196 to 1145 BCE), showing smallpox lesions, e.g., on the bridge of his nose (https://en.wikipedia.org/wiki/Ramesses_V#/media/File:Ramses_V_mummy_head.png).

Around 12,000 years ago, small family/clan groups of humans abandoned nomadic hunting and gathering to settle down in stable locations, cultivating crops and raising domestic animals for food, labor, and clothing (the Neolithic revolution). For the first time, humans and newly domesticated animals were living together in complicated ecosystems of villages, towns, cities, and pasturages. Under conditions of intense human-animal proximity and environmental alterations, enzootic and zoonotic diseases appeared. The agents of measles, smallpox, tuberculosis, gastric cancer (caused by Helicobacter pylori), and many other pandemic diseases evolved from animal pathogens that switched hosts to become human infectious agents. As human populations continued to expand, these agents were able to initiate epidemics and pandemics (Fig. 1). Some of the biblical plagues were probably emerging infectious diseases (5, 6). The preserved mummy of Pharaoh Usermaatre Sekheperenre Ramesses V clearly shows smallpox lesions (Fig. 2), indicating that fatal smallpox epidemics prevailed more than 3,000 years ago (7). At some point, smallpox spread pandemically over most of the world, sparing the Western Hemisphere for millennia, up to the 16th century, when the first known epidemic occurred there in 1520. Until its eradication was declared in 1980, smallpox killed untold millions over at least 3 millennia. Even diseases now commonly dismissed as benign, like measles, killed millions; little more than a century ago, measles case fatality reached as high as 30%, especially in indigenous populations with vitamin A deficiency (2).

Heralding the end of Greece’s Golden Age, the explosive “plague of Athens” (430 to 425 BCE) was perhaps the first recorded pandemic: it spread over much of the world known to the Greeks, including the Mediterranean and northern Africa (8). Although the cause of the Athenian plague has not been identified (anthrax, bubonic/pneumonic plague, smallpox, and typhus are leading candidates), it was the first disease investigated and described using clinical and epidemiological approaches. It remains today a benchmark for pandemic comparisons.

**FIG 3:**
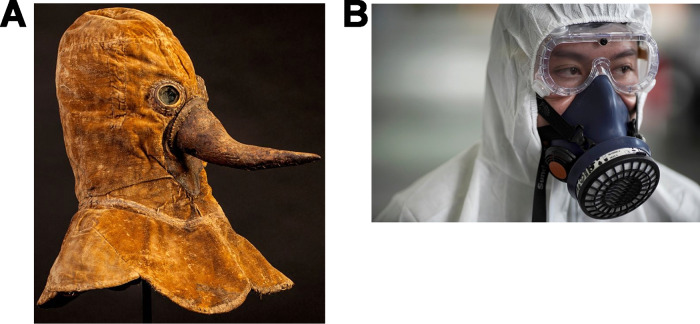
Fighting plagues (A) in the 17th century (https://www.dhm.de/blog/2017/07/27/beaky-plague-protection/) and (B) in 2020 (caused by SARS-CoV-2 [https://www.nst.com.my/world/world/2020/01/560477/only-high-quality-masks-can-defend-against-coronavirus,%20image%20used%20by%20permission%20of%20Reuters%20News%20Agency, image used by permission of Reuters News Agency]).

Since the Athenian plague, there has been a steady stream of new pandemics of even greater mortality (Table 1) (2). Confronting them and then quickly forgetting the lessons that they left behind have become a recurring theme in human existence. The repetitive nature of our struggles to combat these diseases is illustrated in countless history books and plague tractates, with sometimes striking similarities in avoidance and control strategies across the centuries (Fig. 3).

## WHAT IS A PANDEMIC?

“Pandemic” has never been a scientific term but rather a subjective popular term. In use since the mid-1600s, the word “pandemic” (or “pandemick”) was at first so imprecise that it could mean different, even contradictory, things in different contexts (3). At its most specific, it conveyed the vague notion of an impressively large epidemic, and its Greek roots, “pan” (all) and “demos” (people), reflect its widespread nature. “Epidemic” is often translated from the Greek as “that which is upon the people,” i.e., a high-incidence or widely prevalent condition, and is most frequently employed when there is rapid temporal and geographical spread. Following the sudden emergence of global influenza in 1889, the term “pandemic” acquired, and as of today officially retains, the narrower meaning of a disease “…occurring widely throughout a region, country, continent, or globally” (3). “Pandemic” has also been subcategorized into transregional (widespread within a continent or other large region), interregional (involving two or more regions), and global (4) categories. In practice, “pandemic” and “epidemic” are most often applied to infectious diseases, largely replacing such historical terms for emerging infections as *loimos*, *peste*, pestilence, and plague (9) (in situations where “plague” is used generically, rather than in specific reference to bubonic/pneumonic plague caused by Yersinia pestis).

What lessons have we learned from this long history of pandemics, and how do they relate to the current situation with COVID-19?

## HUMAN BEINGS ARE THE ULTIMATE CAUSES OF PANDEMICS

Pandemics are caused by specific organisms, but these same organisms, or their ancestors, have in almost all cases been around us for millennia without causing pandemic harm. As noted above, it was the historical congregation of humans and domestic animals in villages and cities that provided the opportunity for ancestral organisms to switch their hosts to humans and cause human smallpox, measles, and other diseases. While these infectious agents originated in once-wild animals that were then domesticated, our growing ecological footprint seems currently to be leading to an exponential rise in the spillover of other microbes directly from wildlife to people (10).

## DEFORESTATION, AGRICULTURAL INTENSIFICATION, URBANIZATION, AND ECOSYSTEM DISRUPTION BRING PEOPLE INTO CONTACT WITH WILDLIFE AND THEIR POTENTIALLY ZOONOTIC PATHOGENS

These activities have led to emerging diseases as diverse as hemorrhagic fevers, Nipah infection, and hantavirus infections (11–13). Since 1999, China’s numerous live-animal markets have arguably led to three important epidemics and now, a pandemic, although the ultimate origins of pandemics can rarely be known with certainty. The emergence of deadly “bird flu” associated with the poultry-adapted influenza A viruses known as H5N1 and H7N9 have killed over a thousand people, SARS killed 774 and came close to causing a global pandemic in 2002 and 2003, and now in 2019 and 2020, SARS-like SARS-CoV-2 is causing our newest pandemic, COVID-19. One seemingly simple human behavior, establishment of multiple large live-animal markets in a populous region, or at least the greatly increased human-wild animal contacts that such markets represent, has within 2 decades caused the emergence of four fatal zoonotic diseases, including one barely prevented near pandemic, and one we have clearly failed to prevent.

## WHEN PEOPLE TRAVEL, GERMS TRAVEL (14); WHEN GERMS TRAVEL, PANDEMICS BECOME POSSIBLE

Beginning around 1320, the “Black Death” followed trade routes from what is now Mongolia and China, across Asia, and into Europe (1347 to 1348). Likewise, cholera spread along travel routes from India to Europe in 1831 to 1833, 1845, 1866, and 1892; its 1831 approach was reported in the media in “real time,” forcing the realization, even without a concept of microbial infection, that cholera advanced exactly as fast as human travel and therefore was undoubtedly a human-caused pandemic (2). In its earliest days, the United States was struck by recurring deadly epidemics along the Eastern seaboard caused by the “American plague” (yellow fever). Between 1793 and 1798, major port cities, such as Boston, MA, and New York, NY, were repeatedly affected. In the nation’s capital, Philadelphia, PA, 10% of residents died; President Washington and the Congress fled the city. Historians have claimed that the national terror caused by these epidemics, now known to have resulted from annual importations of yellow fever virus and its vector, Aedes aegypti mosquitoes from Caribbean trading ships, forever changed the national character of the American people.

HIV is believed to have emerged at some time between 1880 and 1920, but it did not become pandemic until 1981, when the global population size had expanded, human movement had become more geographically extensive, and complex facilitative human behaviors (e.g., transnational road building and truck routes, leading to travel-related prostitution, and affordable international air travel) had been more fully developed. Influenza pandemics have been noted to follow human population movement since 876 AD and in the past 130 years have been repeatedly shown to follow rail, ship, and air routes (15, 16).

The Aedes aegypti-borne diseases (yellow fever, dengue, chikungunya, and Zika) are all associated with human crowding/imperfect sanitation, peri-domestic water storage, exportation of vector mosquitoes, and human development of novel mosquito breeding sites, such as discarded rubber tires. These four arboviral diseases have all exploded in recent decades, the delayed result of the emergence and adaptation of a single mosquito species in response to water storage behaviors of humans beginning more than 5,000 years ago and which are being greatly amplified today (17). The unwitting spread of microbes by humans, a process termed “pathogen pollution,” accelerates the geographic spread of emerging diseases and their impact on morbidity and mortality. In a world now dominated by a globalized economy that depends on international travel and trade, such disease spread has led to significant economic losses, e.g., $30 to 50 billion for SARS and multiple hundreds of billions of dollars (so far) for COVID-19 (10).

The reality that humans are the ultimate cause of pandemics is demonstrated most tragically by what historian Alfred Crosby has referred to as the “Columbian exchange” (18). After the first voyage of Columbus to the Americas in 1492, syphilis was apparently brought back to Europe. Even more devastating consequences quickly followed. Europeans soon brought smallpox, measles, and other previously unknown diseases to the New World, wiping out millions of native peoples, e.g., the infamous *hueyzahuatl* epidemic of 1520 (Table 1), which killed an estimated 3.5 million (historians believe the epidemic was caused mostly if not entirely by smallpox, with a possible role for measles as well). During the next several hundred years, all over the Americas, countless millions of native people died from these and other imported diseases. Beginning in the 1700s, the tragedy was extended by explorers to the Pacific islands. The near extinction of native peoples over half of the globe occurred on a scale so massive that it was never adequately measured. The age of exploration might more appropriately be called the age of global microbial devastation.

## EXPECT THE UNEXPECTED

**FIG 4:**
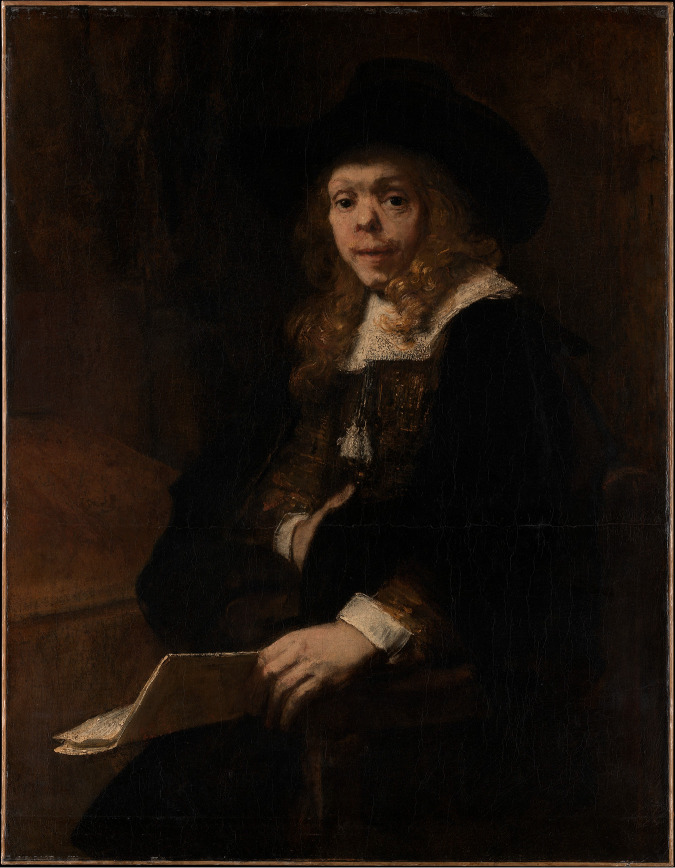
1665 portrait of renowned painter, poet, and public intellectual Gérard de Lairesse (1641 to 1711), by Rembrandt Harmenszoon van Rijn (1606 to 1669). Lairesse’s facial deformities caused him to be shunned by some contemporaries; they are now thought to have resulted from congenital syphilis. (https://www.metmuseum.org/art/collection/search/459082; public domain.)

It has not, so far, been possible to predict the exact timing, place of origin, or clinical-epidemiologic features of any of the recent pandemics. No explosive sexually transmitted disease had ever been seen in Europe at the time that the syphilis pandemic appeared suddenly, in the late 15th century. The horrifying gummatous deformities (Fig. 4) and tragic deaths characteristic of the first decades of the pandemic were likewise unprecedented (19). Four centuries later, the HIV/AIDS pandemic was just as shocking in its ability to cause high fatality and tragic deaths, this time in association with multiple human-to-human modes of transmission (e.g., sexual transmission, needle sharing, blood product transfusion, maternal transmission), significantly complicating control efforts. More than a millennium of at least 20 pandemic influenza recurrences (at least one every 57 years, and since 1700 AD, one every 32 years [4]) has surprised us in each instance, in some cases, e.g., 1918, with extraordinary mortality and inexplicable epidemiologic features (4, 16). Strong human reactions to the unexpected and frightening consequences of pandemics, including reactions to the highly fatal Ebola and the tragic deformities of babies during the Zika pandemic, have characterized almost all pandemics, and now we are experiencing shock at the overwhelming of hospitals and the hastily dug mass graves associated with COVID-19.

However, science is beginning to provide hope that we can predict some aspects of pandemic emergence and hopefully begin to lower the risk. Tracking past pandemic origins allows us to identify the underlying causes of emerging diseases and hot spots where they are most likely to originate, although these may be very large regions. Analyzing host-virus relationships allows us to identify wildlife species that carry the highest risk of as-yet-undiscovered viruses (20) and to estimate how many of these there are in wildlife (21). Analyses of air travel pathways provide real-time data to anticipate the likely spread of novel diseases once they have gained a foothold in the human population. Much remains to be done, but these efforts provide the first steps to what may one day become a preventive approach to pandemic emergence. If land use change and agricultural intensification drive disease emergence, future programs to reduce human-wildlife contact around these activities may reduce the risk of future pandemics. In addition, mounting successes in biomedical science are chipping away at existing infectious diseases; smallpox and the veterinary disease rinderpest have been eradicated, and other diseases, such as polio, dracunculiasis, measles, and rubella, among others, are current eradication targets. We now cure once-fatal bacterial diseases with antibiotics, have developed many effective vaccines, have an antiviral cure for hepatitis C, and use antiviral cocktail therapy to keep millions of HIV-infected persons alive and healthy. Nature may be relentless, but science continues to push back.

## PUBLIC HEALTH AND CIVIL ORGANIZATIONAL MANAGEMENT ARE CRITICAL TO PANDEMIC CONTROL

Even in our modern era of drugs and vaccines, the most important first steps in pandemic control are preventive and educational. Infection-specific drugs and vaccines are rarely available at the outset and may not be available for years. When they become available, stockpiles may be insufficient, especially in the developing world. Moreover, initially, diagnostics may be unavailable or nonspecific, and there may be too few medical providers and facilities. An influenza or a COVID-19 pandemic as fatal as the 1918 influenza pandemic, even without adjustment for the significantly older U.S. population age, might require, over a period of 2 to 3 months, as many as 2 to 4 million fully staffed intensive care units (ICUs) with ventilators, drugs, and supplies. Although estimates vary widely, the current U.S. surge capacity has been said to be around 46,500 ventilator-equipped ICUs. Public health efforts, including those organized by local and state health departments, and those provided by government, industry, and nongovernmental organizations (NGOs) (22) are by far the most critical components of early pandemic responses. These must be greatly strengthened.

## WHAT DOES PANDEMIC HISTORY TELL US ABOUT CONFRONTING COVID-19?

Every pandemic is different. Roughly 18 weeks into the COVID-19 pandemic (17 April 2020), we remain unsure of what lies ahead. Controlling a pandemic can be compared to dancing with an unpredictable leading partner. Neither where the dance is going nor the direction of the next leading step can be known. The trick is to remain alert, flexible, and capable of changing strategy at any moment as the situation itself changes. To complicate matters, the changing situation requires not only good management of uncertainty but good communication about uncertainty to a confused public. In early 2020, we are grudgingly relearning that public health efforts, such as “social distancing,” shuttering of businesses, prevention of mass gatherings, travel restrictions, etc., can be highly effective when adopted early and stringently. Similar lessons were learned centuries ago in earlier pandemics, e.g., during the plague pandemic of the 1340s, which brought about the establishment of quarantine, publication of numerous preventive medicine “plague tractates” to guide personal and public actions, and self-isolation, as described in Boccaccio’s classic *Decameron*, in which young people sequestered themselves in the Florentine countryside to avoid the plague raging in the city.

That China has been able to achieve at least short-term regional control reminds us of the often-unused potential of public health police power. Some other countries/jurisdictions, e.g., Hong Kong, Singapore, and South Korea, have controlled early COVID-19 waves as well. Anecdotal evidence ascribes these successes to early aggressive public health interventions, such as mass PCR testing, contact tracing, and isolation, but more evidence is needed to rule out the biasing effects of ecologic comparisons and to establish the efficacy of specific public health actions. However, other countries with sophisticated public health capacities, e.g., Italy, have not had early success at controlling viral spread, and there is a growing realization that, as is true for many other respiratory viral diseases, “silent spreading” of SARS-CoV-2 by people who are either presymptomatic (incubating), asymptomatic, or exhibiting mild or atypical symptoms is undoubtedly driving the COVID-19 pandemic (23). Confronting these dynamics will be of critical importance. Ever since the late 19th century, U.S. and most Western public health experts have recognized that there is usually far more to be gained by fostering public trust than by threatening public health police power, e.g., by forcibly isolating, quarantining, or preventing travel and movement (24, 25).

Even so, public health control options lie on a continuum from informative/suggestive to coercive; the right balance must always be sought and can be expected to change as the pandemic progresses. Already, in addition to public health agencies, businesses, schools, cultural entities, and government agencies are taking public health actions against COVID-19, including temporary or indefinite closures. So far, the U.S. public seems to be moving in step with recommendations of public health, civic, and industry leaders. Personal, private, and nongovernmental efforts may be definitive. It is critical that such efforts be sustained as the pandemic worsens. We do not know whether the heat and humidity of summer weather in the Northern Hemisphere will slow down COVID-19’s spread, as is the case with influenza, or whether intense COVID-19 circulation will eventually lead to selection of viral variants that escape population immunity, which is also the case for influenza, but we must be ready for all such possible twists and turns.

We must also now ask whether the three unprecedented coronavirus emergences within 17 years are harbingers of a new era in which additional members of the presumably large universe of enzootic coronaviruses will repeatedly emerge to threaten us for the foreseeable future. To begin to answer this question, we need a much more complete characterization of these viruses in nature, not only from identifying viral genomes but also from studying live viruses experimentally. The few dedicated scientists involved in this work over the past decade, including U.S.-Chinese teams working in China, have had to work with minimal funding and against many unhelpful obstacles. Among many important questions to be answered is whether enzootic coronaviruses have shared epitopes that will be cross-protective if presented in vaccines. Such questions can be answered only by robust collaborative research of all aspects of this disease, including virologic, immunologic, clinical, pathological, and epidemiologic studies.

## “WE MUST ALL HANG TOGETHER, OR WE WILL ALL HANG SEPARATELY”

How well we will succeed in mitigating the pandemic of COVID-19 cannot be predicted, but going forward, we must keep an eye on the abundant lessons left us by the histories of past pandemics. We must also take note of what is going on in nature all around us. Other species have not been as lucky as we have been so far. Species of bats, bees, and frogs are now being threatened with extinction by pan- and epizootic diseases; we should not imagine that humans will be exempt from natural laws of microbial evolution (26–28). Both the Plague of Justinian (541 AD) and the Black Death (1347 AD) are believed to have killed large percentages of the global human population known to Western and Middle Eastern scholars of the time. What assurance do we have that something as deadly will not soon appear?

When the COVID-19 pandemic has run its course, whatever the level of devastation it has left in its wake, it will be time to take stock and rethink how we can fix inadequate pandemic defenses. This must be a cooperative global undertaking, because we can expect to face pandemic challenges again and again, and global pandemic threats cannot be managed by national responses. In a densely interconnected world of nearly 8 billion humans, we have no choice but to follow Benjamin Franklin’s revolutionary advice and hang together for the good of all.

Pandemics are nature’s loud wake-up call that we humans are mismanaging our own existence in the complex ecosystem that we have thoughtlessly shaped, within which we live, and upon which our survival depends: planet Earth. We must not only wake up, we must now get up, with energy, and start building a safer future on a healthier planet.

## References

[1] Camus A 1947 La peste. Paris: Gallimard, 1947.

[2] Morens DM, Folkers GK, Fauci AS 2008 Emerging infections: a perpetual challenge. Lancet Infect Dis 8:710–719. doi:10.1016/S1473-3099(08)70256-1. 18992407 PMC2599922

[3] Morens DM, Folkers GK, Fauci AS 2009 What is a pandemic? J Infect Dis 200:1018–1021. doi:10.1086/644537. 19712039

[4] Morens DM, Taubenberger JK 2011 Pandemic influenza: certain uncertainties. Rev Med Virol 21:262–284. doi:10.1002/rmv.689. 21706672 PMC3246071

[5] Dobson AP, Carper ER 1996 Infectious diseases and human population history. Bioscience 46:115–126. doi:10.2307/1312814.

[6] McNeill WH 1976 Plagues and peoples. Doubleday, New York, NY.

[7] Hopkins DR 1983 Princes and peasants: smallpox in history. University of Chicago Press, Chicago, IL.

[8] Morens DM, Littman RL 1994 Thucydides syndrome reconsidered: new thoughts on the plague of Athens. Am J Epidemiol 140:621–628. doi:10.1093/oxfordjournals.aje.a117299. 7942762

[9] Hippocrates. The Genuine Works of Hippocrates. New York: William Wood and Co., 1891.

[10] Jones KE, Patel NG, Levy MA, Storeygard A, Balk D, Gittleman JL, Daszak P 2008 Global trends in emerging infectious diseases. Nature 451:990–993. doi:10.1038/nature06536. 18288193 PMC5960580

[11] Morens DM, Folkers GK, Fauci AS 2004 The challenge of emerging and re-emerging infectious diseases. Nature 430:242–249. doi:10.1038/nature02759. 15241422 PMC7094993

[12] Morens DM, Daszak P, Taubenberger JK 2020 Escaping Pandora’s box—another novel coronavirus. N Engl J Med 382:1293–1295. doi:10.1056/NEJMp2002106. 32101660

[13] Allen T, Murray KA, Zambrana-Torrelio C, Morse SS, Rondinini C, Di Marco M, Breit N, Olival KJ, Daszak P 2017 Global hotspots and correlates of emerging zoonotic diseases. Nat Commun 8:1124–1124. doi:10.1038/s41467-017-00923-8. 29066781 PMC5654761

[14] Markel H 2004 When germs travel: six major epidemics that have invaded America since 1900 and the fears they have unleashed. Pantheon Books, New York, NY.

[15] Taubenberger JK, Morens DM 2009 Pandemic influenza—including a risk assessment of H5N1. Rev Sci Tech 28:187–202. doi:10.20506/rst.28.1.1879. 19618626 PMC2720801

[16] Morens DM, Taubenberger JK, Fauci AS 2009 The persistent legacy of the 1918 influenza virus. N Engl J Med 361:225–229. doi:10.1056/NEJMp0904819. 19564629 PMC2749954

[17] Morens DM, Fauci AS 2014 Chikungunya at the door: déjà vu all over again? N Engl J Med 371:885–887. doi:10.1056/NEJMp1408509. 25029435

[18] Crosby AW 1972 The Columbian exchange: biological and cultural consequences of 1492. Greenwood Publishing Group, Santa Barbara, CA.

[19] Tagarelli A, Piro A 2014 On the illness of Politian (Agnolo Ambrogini, 1454–1494): syphilis at its identification in Europe. J Med Biogr 22:163–171. doi:10.1177/0967772014533044. 24913847

[20] Olival KJ, Hosseini PR, Zambrana-Torrelio C, Ross N, Bogich TL, Daszak P 2017 Host and viral traits predict zoonotic spillover from mammals. Nature 546:646–650. doi:10.1038/nature22975. 28636590 PMC5570460

[21] Carroll D, Daszak P, Wolfe ND, Gao GF, Morel CM, Morzaria S, Pablos-Méndez A, Tomori O, Mazet JAK 2018 The global virome project. Science 359:872–874. doi:10.1126/science.aap7463. 29472471

[22] Navarro JA, Kohl KS, Cetron MS, Markel H 2016 A tale of many cities: a contemporary historical study of the implementation of school closures during the 2009 pA(H1N1) influenza pandemic. J Health Polit Policy Law 41:393–421. doi:10.1215/03616878-3523958. 26921384 PMC5595096

[23] Li R, Pei S, Chen B, Song Y, Zhang T, Yang W, Shaman J 2020 Substantial undocumented infection facilitates the rapid dissemination of novel coronavirus SARS-CoV2. Science 368:489–493. doi:10.1126/science.abb3221. 32179701 PMC7164387

[24] Markel H, Lipman HB, Navarro JA, Sloan A, Michalsen JR, Stern AM, Cetron MS 2007 Nonpharmaceutical interventions implemented by US cities during the 1918–1919 influenza pandemic. JAMA 298:644–654. doi:10.1001/jama.298.6.644. 17684187

[25] Markel H 1997 Quarantine! East European Jewish immigrants and the New York City epidemics of 1892. Johns Hopkins University Press, Baltimore, MD.

[26] Wyatt KB, Campos PF, Gilbert MTP, Kolokotronis S-O, Hynes WH, DeSalle R, Ball SJ, Daszak P, MacPhee RDE, Greenwood AD 2008 Historical mammal extinction on Christmas Island (Indian Ocean) correlates with introduced infectious disease. PLoS One 3:e3602. doi:10.1371/journal.pone.0003602. 18985148 PMC2572834

[27] Daszak P, Cunningham A 1999 Extinction by infection. Trends Ecol Evol 14:279. doi:10.1016/s0169-5347(99)01665-1. 10370265

[28] Schloegel LM, Hero JM, Berger L, Speare R, McDonald K, Daszak P 2006 The decline of the sharp-snouted day frog (*Taudactylus acutirostris*): the first documented case of extinction by infection in a free-ranging wildlife species? Ecohealth 3:35–40. doi:10.1007/s10393-005-0012-6.

